# Efficient conversion of 5-hydroxymethylfurfural to high-value chemicals by chemo- and bio-catalysis

**DOI:** 10.1039/c8ra05308a

**Published:** 2018-09-03

**Authors:** Haian Xia, Siquan Xu, Hong Hu, Jiahuan An, Changzhi Li

**Affiliations:** Jiangsu Provincial Key Lab for the Chemistry and Utilization of Agro-forest Biomass China haxia@dicp.ac.cn +86-25-85428873 +86-25-85427635; School of Chemical Engineering, Nanjing Forestry University Nanjing 210037 China; Dalian Institute of Chemical Physics, Chinese Academy of Sciences Dalian 116023 China licz@dicp.ac.cn

## Abstract

5-hydroxymethylfurfural (HMF) is a very important versatile platform compound derived from renewable biomass. The functionalized molecule with an aldehyde group, a hydroxyl group and a furan ring provides great potential for the production of a wide variety of valuable chemicals. This review highlights the latest advances in the catalytic conversion of HMF into value-added chemicals by some important reactions including (1) aerobic oxidation of HMF into furan-based aldehydes and acids such as 5-hydroxymethyl-2-furancarboxylic acid (HMFCA), 2,5-diformylfuran (DFF), and furandicarboxylic acid (FDCA), (2) reductive amination of HMF to amine, (3) the synthesis of aromatics by Diels–alder reaction followed by a dehydration reaction, (4) catalytic reduction of HMF into 2,5-bis(hydroxymethyl)furan (BHMF), and 2,5-dimethyl furan (DMF), (5) catalytic oxidation of HMF into maleic anhydride, and some other important transformations. The review mainly focuses on the recent progress in bio-catalytic, electrocatalytic, and heterogeneous catalytic transformation of HMF into high value chemicals over the past few years. Moreover, an outlook is provided to highlight opportunities and challenges related to this hot research topic.

## Abstract 2

5-hydroxymethylfurfural (HMF) is a very important versatile platform compound derived from renewable biomass.

## Graphical abstract

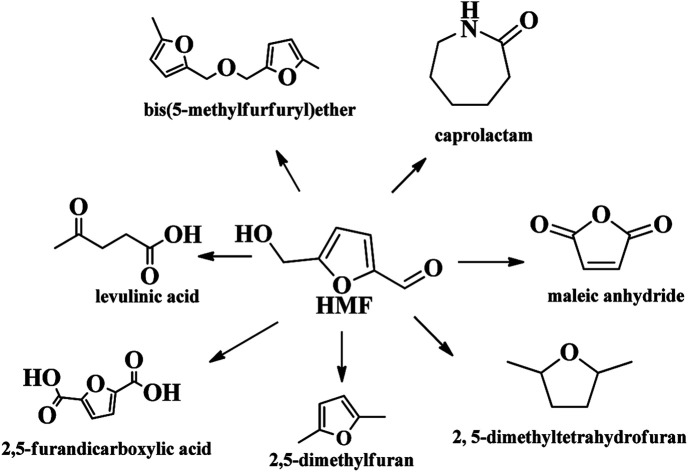

## 1. Introduction

In the 20th century, petroleum, fossils and other non-renewable energy sources have contributed to the continuous progress of society and the rapid development of the economy. However, the contradiction between the diminishing non-renewable resource reserves and the increasing demands for fuels, as well as the environmental pollution caused by overuse and exploitation of fossil resources, has become a huge challenge for mankind.^1^ Faced with this dilemma, people have put forward strategies to search for renewable alternative resources. Biomass is the only renewable organic carbon source in nature, which has the characteristics of being environmentally friendly, having abundant reserves and being low cost, which endow it with unique advantages in producing fuels and industrially important chemicals.^1–3^ Presently, the overall biomass production accessible per annum in the world is around 1 × 10^11^ tons, however, the vast majority are consumed by inefficient technologies and discarded.^4,5^ Therefore, cost effective, efficient and green conversion of biomass into sustainable fuels and chemical products is particularly significant.

**Scheme 1:**
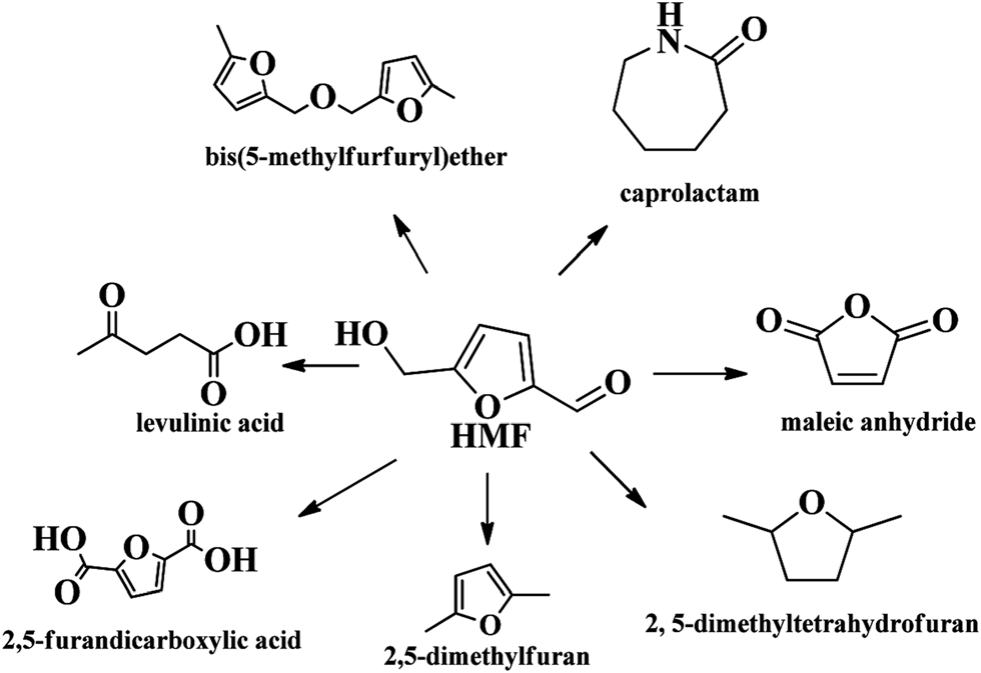
HMF as a platform compound for diverse reaction.

Amongst various valuable compounds derived from biomass, HMF is identified to be a top building block chemical.^6^ HMF possesses a very versatile chemical activity, making it possible to be further transformed into value-added chemicals including 2,5-furandicarboxylic acid (FDCA),^7,8^ 2,5-dimethyl furan (DMF),^9,10^ levulinic acid^11^ and other chemicals through oxidation, hydrogenation, hydrolysis, *etc.* (Scheme 1).^12–14^ Great progresses have been made in the valorization of HMF into chemicals in recent years. For example, FDCA, a very important monomer, which has been commercially produced through a homogenous catalytic system by Avatium company.^15^ However, there remains a huge challenge for the production of FDCA from HMF by using heterogeneous catalyst due to the stability of the catalysts and low yield upon using high concentration of substrate HMF.^15,16^ For DMF, its high yield (>99%) is readily obtained by various heterogeneous catalysts, but the recyclability of the catalysts and expensive HMF cost limit the large scale commercial product.^17^ Although there are some challenges for the commercial production of HMF-based chemicals, HMF is still deemed as a key intermediate that bridges bio-based products and carbohydrate chemistry, efficient production and rational utilization of bio-based HMF is envisaged to be promising to achieve sustainable biorefineries. It is urgent to decrease the production cost of HMF by using cheap material and to overcome the bottleneck question for low yield of HMF, especially for the use of lignocellulose as starting material in order to realize large scale production of bio-chemicals derived from HMF.

**Scheme 2:**
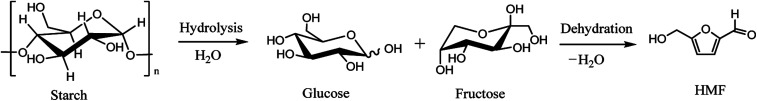
Schematic illustration of the transformation of starch to HMF.

Dehydration of monosaccharides and polysaccharides, which are widely distributed in biomass, is the main synthetic pathway of HMF.^18^ Various substrates are used for the production of HMF: hexoses themselves, oligosaccharides as well as cellulose^19–21^ and the raw biomass such as wheat straw (see in Scheme 2).^22^ It is generally accepted that the production of HMF from biomass must include the acid-catalyzed hydrolysis of biomass to produce hexose in the first step. In the second dehydration step, starts from ketohexose (*e.g.* fructose) is more efficient and more selective than from aldohexose (*e.g.* glucose), due that the structure of aldohexose is very stable and it enolyses in a very low degree, while the enolisation is a determining factor for the HMF formation from hexoses.^23,24^ Over the past few years, many excellent reviews about the production of HMF from carbohydrates have been documented.^3,14,25,26^

Efficient utilization of HMF into chemicals mainly proceeds through homogeneous-, heterogeneous-, bio-, and electrochemical catalysis techniques. These catalysis techniques have their own advantages and drawbacks, whereas heterogeneous- and bio-catalysis are more commonly used in the transformation of HMF into chemicals compared to other strategies. Besides bio-chemicals, the generation of bio-fuels with HMF as a starting materials is an very important utilization pathway, including jet fuel hydrocarbon,^27–30^ ethers,^17,31^ and so on. Many excellent work and reviews on the synthesis of bio-fuels has been extensively reported.^3,14,17,32^ Therefore, in this review, we only focuses on the transformation of HMF into bio-chemicals. To the best of our knowledge, no review focused on bio-catalytic, heterogeneous, and their hybrid catalytic conversion of HMF into chemicals has been reported although there are some excellent reviews about the conversion of HMF into high value chemicals only by a single chemo-^33^ or bio-catalysis.^34^ The present review summaries the latest advances in the production of chemicals from HMF regarding heterogeneous chemo-catalysis, bio-catalysis and their hybrid approaches. In addition, a prospect was provided to highlight the challenges and opportunities for the utilization of HMF as sustainable chemicals and fuels.

## 2. Conversion of HMF to high-value downstream products

### 2.1 Oxidation of HMF to 2,5-furandicarboxylic acid (FDCA)

In the past, oxidation of HMF to FDCA can be performed by homogeneous, and electrocatalysis, and heterogeneous catalysis. In the recent year, bio-catalysis has attracted increasing attentions in the oxidation of HMF into FDCA. If homogeneous catalysis was used in the oxidation of HMF into FDCA, the addition of base is acquired, whereas separation of FDCA will result in a high operating cost to purchase the required acid to dispose of the resulting waste salt. Therefore, many environmentally friendly and economical methods are currently bedding exploited for the oxidation of HMF to FDCA.

#### 2.1.1 Heterogeneous catalysis

FDCA molecule contains two carboxyl groups in the side chain of furan ring, which is structurally similar to terephthalic acid (PTA), the principal precursor to polyethylene terephthalate (PET) molecule that used in making plastics and a wide array of industrial products. Therefore, FDCA is considered an excellent substitute for terephthalic acid and can be used in the synthesis of polymers, especially polyethylene furanoate (PEF).^35^

**Scheme 3:**
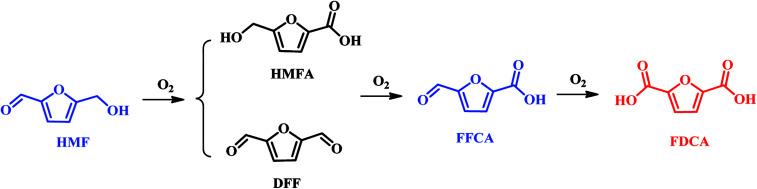
Reaction pathway for aqueous HMF oxidation.

The catalytic conversion of HMF to FDCA is a complex oxidation process, which forms several intermediate products such as 5-hydroxymethyl-2-furancarboxylic acid (HMFCA), 2,5-diformylfuran (DFF), 5-formyl-2-furancarboxylic acid (FFCA), as illustrated in Scheme 3. A variety of catalysts, including enzyme, homogeneous metal salts, stoichiometric oxidants and heterogeneous metal or metal oxide have been explored in the oxidation of HMF to FDCA.^36–39^ The corresponding results are summarized in Table 1. Among them, noble metal catalysts such as Pd, Pt, Ru and Au based catalysts exhibit excellent catalytic activity under mild conditions.^8,40,41^ Davis *et al.* achieved 79% and 68% FDCA yields with HMF fully conversion at 22 °C using Au/TiO_2_ and Pt/C catalysts, respectively.^40^ In the work of Steinfeldt and co-workers, high FDCA yield of 90% was obtained using Pd/ZrO_2_/LaO_2_ as a catalyst in aqueous media.^8^

**Table 1:** Different catalysts for the conversion of HMF into FDCA

Entry	Catalyst	Base	*T* (°C)/*t* (h)	Solvent	HMF con. (%)	FDCA yield (%)	Ref.
1	Au/TiO_2_	NaOH	22/22	H_2_O	100	79	40
2	Pt/C	NaOH	22/22	H_2_O	100	68	40
3	Pd/ZrO_2_/LaO_2_	NaOH	90/4	H_2_O	100	91	8
4	Au–Pd/AC	NaOH	60/2	H_2_O	100	95	48
5	Mn/Fe (3 : 1) mixed oxides	NaOH	90/24	H_2_O	93	32	7
6	Merrifield resin-Co-Py	—	100/24	CH_3_CN	96	91	50
7	NNC	K_2_CO_3_	80/48	H_2_O	100	80	50
8	Pt/C–O–Mg	—	11 012	H_2_O	100	97	52
9	Pt/CNT	—	95/12	H_2_O	100	98	52
10	Ru/CTF	—	140/1	H_2_O	99	78	51
11	Ru/MnCo_2_O_4_	—	12 010	H_2_O	100	99	53

It has been revealed that Pt and Pd nanoparticles are more active for the oxidation of an alcohol side chain, whereas Au nanoparticle is more active for the oxidation of an aldehyde side chain.^15,37,42,43^ It should be noted that the oxidation performances toward HMFCA of these metal nanoparticles depend on the structure of the catalysts including the support type, the size of nanoparticles, the facet-effect, *etc.*^15,38,40^ In other words, the aerobic oxidation of HMF is structure-sensitive reaction. To investigate the facet effect of Pd nanocrystals on the aerobic oxidation of HMF, Liu *et al.* synthesized single-crystalline Pd nanooctahedrons and nanocubes enclosed by (111) or (100) facets with controlled size using polyvinylpyrrolidone (PVP) as a capping agent.^44^ They found that the size-dependent effect of these Pd nanocrystals could only be attributed to the different Pd dispersions. Interestingly, they also demonstrated that Pd nanooctahedrons enclosed by (111) facet showed remarkably enhanced catalytic activity compared to Pd nanocubes enclosed by (100) facets for the aerobic oxidation of HMF, which is attributed to the reason that Pd nanooctahedrons has a lower energy in the alcohol oxidation step from HMFCA to FFCA in comparison with Pd nanocubes.^44^

In comparison with the monometallic catalyst, the use of bimetallic catalysts is an interesting strategy as it is able to enhance the activity and the product yield by modifying the electronic structure and the synergic effect.^41,45–47^ Prati *et al.* reported that Pd-modified Au supported on active carbon (AC) can act as an efficient and durable catalyst for the aerobic oxidation of HMF to FDCA. A series of bimetallic Au–Pd/AC with different metal ratios achieved FDCA yield higher than 95% with 100% HMF conversion.^48^ Apparently, the results demonstrated that the bimetallic catalyst was beneficial to improve the activity, the yield of FDCA, and the stability of the catalyst.

**Scheme 4:**
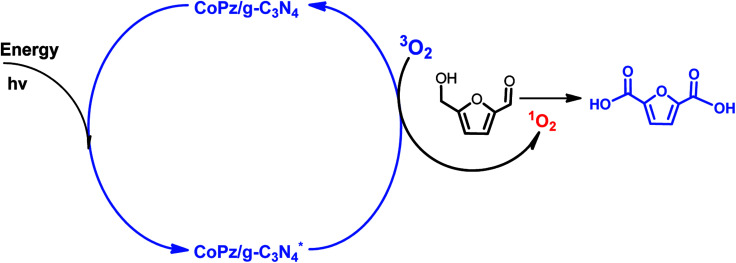
Possible mechanism of the photocatalytic oxidation of HMF into FDCA with the CoPz/g-C_3_N_4_ catalyst.

Despite of these progresses, most of the above mentioned catalytic systems use expensive noble metal and some of them need excessive base, these drawbacks hamper their potential for practical application. In order to address the problems, non-noble metal catalysts and metal-free catalysts in the absence of base have been developed for the conversion of HMF to FDCA. For instance, Mn/Fe (3 : 1) mixed oxides,^7^ merrifield resin supported Co(ii)–meso-tetra(4-pyridyl)-porphyrin (merrifield resin-Co-Py)^49^ and nitrogen-doped nanoporous carbon (NNC)^50^ all exhibited high activity in the oxidation of HMF. It is well known that base plays an important role in the production of FDCA from HMF, and hydroxide ions intend to promote O–H and C–H bond activation of the alcohol side chain of HMF and then add directly to aldehyde intermediates to eventually form acid products.^40^ The employment of numerous inherently solid base catalysts avoids the addition of homogeneous base. As an example, Palkovits *et al.* reported that Ru clusters supported on covalent triazine frameworks (Ru/CTF) afford 78% FDCA yield at 140 °C under 20 bar air without a homogeneous base.^51^ Pt/C–O–Mg catalyst gave the yield of FDCA up to 97% under the optimal reaction conditions.^43^ Moreover, without the addition of a base, FDCA yield of 98% was obtained over functionalized carbon nanotubes (CNTs) supported Pt nanoparticles (Pt/CNTs) at 95 °C for 14 h.^52^ Kim and co-workers also reported that the MnCo_2_O_4_ spinels supported Ru catalyst (Ru/MnCo_2_O_4_) was exploited to afford FDCA with an excellent yield of 99% in the absence of base.^53^ Very recently, Zhang *et al.* developed a novel CoPz/g-C_3_N_4_ photocatalyst for the selective oxidation of HMF into FDCA under simulated sunlight with molecular O_2_ in air.^54^ They proposed that ^1^O_2_ species are the active oxygen species responsible for the selective oxidation of HMF, as illustrated in Scheme 4.

Although much effort has made in the aerobic oxidation of HMF into valuable chemical, there still remain a lot of challenges such as the development of more efficient non-noble metal catalyst and more green synthesized strategy without the addition of inorganic base.

#### 2.1.2 Bio-catalysis

As mentioned above, chemo-catalytic oxidation of HMF into FDCA generally requires the addition of inorganic base such as NaOH, NaHCO_3_, *etc.*, thus leading to environmentally unfriendly processes. Bio-catalysis may be a potential alternative for oxidation of HMF into FDCA. The enzymatic full oxidation of HMF into FDCA is not trivial because it involves three successive oxidation steps. Several promising bio-catalytic examples have emerged over the past few years.

A strategy is the combination of enzymes (or with chemo-catalyst) in multistep processes to carry out tandem oxidations route into FDCA. Guajardo *et al.* reported the oxidation of DFF with immobilized TEMPO, followed by a lipase-mediated with the assistance of peracid for further oxidation of DFF to FDCA.^34^ Carnell *et al.* used a combination of galactose oxidase with xanthine oxidoreductase to afford FDCA in promising productivities of 18 g FDCA L^−1^ d^−1^.^55^

Apart from those multistep methods, the development of a single enzyme that catalyzes the triple oxidation of HMF into FDCA is more attractive. Fraaije *et al.* identified a HMF oxidase (HMFO) which is active for the oxidation of HMF and related compounds.^56^ It was found that HMF oxidase (HMFO) is capable of oxidizing 5-(hydroxymethyl)furan-2-yl-methanol into FDCA, and can also produce FDCA from HMF with high yield at ambient temperature and pressure. The underlying mechanism presents that HMF oxidase (HMFO) acts on alcohol groups only and depends on the hydration of aldehydes for the oxidation reaction to produce FDCA.

#### 2.1.3 Electrocatalytic oxidation of HMF into FDCA

Electrochemical oxidation has been regarded as a clean synthetic method because it eliminates the use of O_2_ or other environmentally unfriendly chemical oxidants. In 1991, the electrochemical oxidation of HMF into FDCA was first reported, which afforded a FDCA yield of 71% after 4 h. Recently, Choi *et al.* used electrochemical oxidation in acidic media by using a Mno_*x*_ anode to carry out the oxidation HMF of FDCA, and a FDCA yield of 53.8% was obtained.^57^ In addition, hierarchical Ni–Co based transition metal oxide catalyst^58^ and high surface area nickel boride^59^ were also used for the electrochemical oxidation of HMF to FDCA, and a high faradaic efficiency of close to 100% towards the oxidation of FDCA with a yield of >90% were achieved.

### 2.2 Catalytic hydrogenolysis of HMF to 2,5-dimethylfuran (DMF)

Amongst various valuable compounds derived from biomass, DMF, which is currently produced predominantly from petroleum resource, have received extensive attention because they are good biofuel candidates and important intermediates in chemical industry. For instance, as a fuel replacement, DMF contains ideal boiling point (92–94 °C), high energy density (30 kJ cm^−3^), and high research octane number (RON = 119).^13^ It is also an intermediate for *p*-xylene, one of the highest bulk chemicals presently derived from petroleum.

The results of HMF hydrogenolysis to produce DMF in recent years are summarized in Table 2. A variety of hydrogenolysis catalysts have been reported for the production of DMF from HMF, such as Pd, Pt and Ni based catalysts. In the work reported by Wang *et al.*, a 98% of DMF yield was obtained in the 1-BuOH with PtCo@HCS catalyst at 180 °C.^60^ Huang *et al.* reported that Ni–W_2_C/AC was used as a catalyst to obtain 96% DMF yield in THF.^61^ Rauchfuss and co-workers used formic acid as hydrogen donor and Pd/C as the catalyst for the production of DMF from HMF, affording an excellent yield (>95%).^62^ However, to obtain high DMF yields, formic acid and sulfuric acid must be used simultaneously for these catalysts, which is not environmentally friendly. Subsequently, in the absence of formic acid, Chidambaram and Bell obtained a 32% DMF yield with 47% of HMF conversion over Pd/C in ionic liquids, which avoided the use of unfriendly additives.^63^ But the lower solubility of hydrogen in ionic liquids significantly restrained the conversion efficiency, which is a drawback of this method. Recently, the application of supercritical carbon dioxide–water combined with Pd/C improved mass transfer and turned into a green DMF production pathway, affording a DMF yield of 100%.^64^

**Table 2:** Catalytic hydrogenolysis of HMF into DMF

Entry	Catalyst	Solvent	H_2_ source	*T* (°C)/*t* (h)	HMF con. (%)	DMF yield (%)	Ref.
1	PtCo@HCS	1-BuOH	H_2_ (10 bar)	180/2	100	98	60
2	Ni–W_2_C/AC	THF	H_2_ (10 bar)	180/3	100	96	61
3	Pd/C	THF	Formic acid	150/2	100	>95	62
4	Pd/C	[EMIM]Cl-MeCN	H_2_ (62 bar)	120/1	47	32	64
5	Pd/C	ScCO_2_–H_2_O	H_2_ (10 bar)	80/2	100	100	64
6	CuRu/C	1-BuOH	H_2_ (6.8 bar)	220/–	–	70	13
7	Ru/Co_3_O_4_	THF	H_2_ (7 bar)	130/24	100	94	67
8	Ru-HT	2-Propanol	H_2_ (10 bar)	220/4	100	58	69
9	Cu–PMO	Sc methanol	MeOH	260/3	100	48	69

Besides Pd and Pt-based catalysts, Cu and Ru-based catalysts also have been shown to preferentially catalyze HMF C–O bond hydrogenolysis that minimize aromatic hydrogenation.^65^ A pioneering work reported by Román-Leshkov *et al.* developed a catalytic process for the production of DMF from fructose, bimetallic CuRu/C catalyst provided 70% DMF.^13^ However, this CuRu/C catalyst showed poor tolerance to the presence of chloride ions. A similar catalytic system was reported using non-noble bimetallic Cu–Co catalysts supported on CeO_2_, ZrO_2_, and Al_2_O_3_ for the selective hydrogenolysis of HMF to DMF. High selectivity of 78% was obtained.^66^

The emergence of various stable and reusable catalysts which are not susceptible to chloride ions effectively addressed this challenge. Wang *et al.* prepared Ru/Co_3_O_4_ catalyst that maintained high activity over five cycles, resulting in up to 94% DMF yield under mild reaction conditions.^67^ Nagpure *et al.* used a reusable Ru doped hydrotalcite (HT) catalyst also obtained a modest yield of 58% for the formation of DMF.^68^ Hansen and co-workers employed a stable Cu-containing mixed metal oxide for the catalytic hydrogenation of HMF to achieve 48% DMF yield in supercritical methanol.^69^

### 2.3 Catalytic reduction of HMF into 2,5-bis(hydroxymethyl)furan

#### 2.3.1 Heterogeneous catalysis

2,5-Bis(hydroxymethyl)furan (BHMF) is the hydrogenation product of the aldehyde group in HMF and is a versatile platform molecule for the generation of polymers, drugs, and crown ethers, *etc.* BHMF can be produced mainly by chemical reduction of HMF over noble metal catalysts with molecular H_2_.^70^ It is very expensive for noble metal catalysts such as Pt/MCM-41 and the use of molecular H_2_. In addition, the Cannizzaro reaction was employed in the transformation of HMF to BHMF, but an equimolar byproduct HMFCA was also produced.^71,72^

Recently, the catalytic transfer hydrogenation of HMF by Meerwein–Ponndorf–Verley reduction has been developed and provided a better alternative for the hydro-upgrading of HMF.^73–75^ Formic acid, 2-propanol, and methanol are commonly used as *in situ* hydrogen sources for the hydrogenation of biomass-derived platform molecular. Lin *et al.* developed an efficient process for the catalytic hydrogenation of HMF to BHMF using ethanol as both hydrogen source and solvent on cheap ZrO(OH)_2_.^73^ A high BMHF selectivity up to 88.9% with a HMF conversion of 94.1% were achieved at 423 K in 2.5 h. The external H_2_-free process is cost-efficient due to the usage of non-noble metal in the absence of external H_2_.

#### 2.3.2 Bio-catalysis

Recently, a novel highly HMF-tolerant yeast strain *Meyerozyma guilliermondii* SC1103 was isolated and was used for bio-catalytic whole-cell reduction of HMF into BHMF.^76^ A high BHMF yield of 86% with excellent selectivity of >99% were afforded by using 100 mM HMF as a substrate within 12 h in the presence of 100 mM glucose. He *et al.* also reported that highly HMF-tolerant recombinant *E. coli* CCZU-K14 whole cells can catalyze the transformation of HMF into BHMF with 90.6% yield under the optimum reaction conditions.^77^ Besides the biosynthesis of BHMF, *E. coli* CCZU-K14 also exhibited potential in biological detoxification of lignocellulosic feedstock-derived hydrolysates containing HMF and its derivatives owing to high HMF-reducing efficiency.^77^ Overall, the proof-of-concept of bio-reduction is promising, and further fundamental research as well as techno-economic assessment would be necessitated.

### 2.4 Conversion of HMF to aromatics

**Scheme 5:**
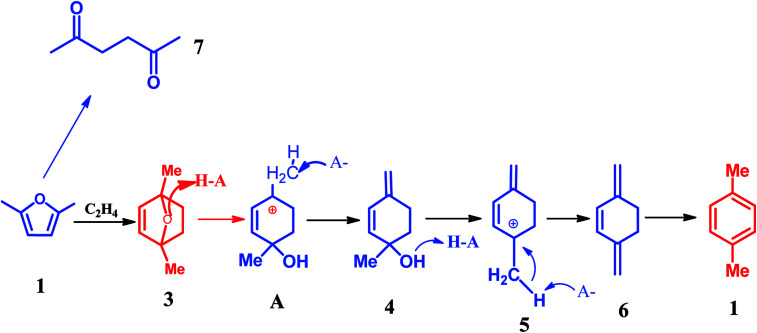
Detailed reaction network of DMF and ethylene at 528 K.^87^

Recently, Dauenhauer,^78–80^ Davis,^81^ Toste,^82^ Tsang^83^ and Zhang,^84,85^ and many other groups^86,87^ reported an interesting route for the synthesis of aromatics from bio-based furan compounds such as 5-hydroxymethylfurfural (HMF), 2,5-dimethylfuran (DMF), 2-methylfuran (MF), furfural. This strategy is of extraordinary significance because it provides the possibility of producing a valuable commodity chemical from lignocellulosic biomass.^79,81,87–91^ It is generally aware that this route undergoes two steps, namely, the Diels–Alder cycloaddition and subsequent dehydration, the detailed reaction network is illustrated in Scheme 5.^92^ As shown in Scheme 5, the Diels–Alder reaction of DMF and ethylene produces oxa-norbornene intermediate 2. Dehydration of intermediate 2 requires an acid catalyst such as zeolite. It was reported that the Diels–Alder reaction of DMF and ethylene is enhanced through the confinement effect inside the zeolite pores.^79^ Protonation of ether 2 and subsequent ring-opening gives allylic cation 3. Deprotonation of 3 at the exocyclic methyl group is more possible due to the least sterically encumbered position adjacent to the cations, which leads to the production of 4. Subsequently, the alcohol 4 further occurs the protonation followed by dehydration results in the highly stabilized pentadienyl cation 5. Finally, the cation 5 is subjected to deprotonation to form the target product *p*-xylene.^87^

Williams *et al.*, reported that a 75% selectivity toward *p*-xylene has been obtained using H–Y zeolite as the catalyst for the cycloaddition of ethylene and DMF and subsequent dehydration with an aliphatic solvent at 300 °C.^93^ Kim *et al.* used mesoporous beta zeolite with a nanosponge-like morphology to catalyze the reaction of DMF with ethylene and produced *p*-xylene with a yield of 80%.^94^ Its appreciable yield is due to the fact that the external surfaces and internal micropores of the catalyst possess a large number of Brønsted acid sites. In addition to the traditional zeolite catalyst, other novel heterogeneous catalysts have also been applied to cyclization of DMF to *p*-xylene. Xin and Zhang reported an original route for the direct synthesis of *p*-xylene from 2,5-dimethylfuran catalyzed by scandium(iii) triflate (Sc(OTf)_3_) in 1-ethyl-3-methylimidazolium bis(trifluoromethylsulfonyl)imide ionic liquid under mild conditions.^95^ In this process, the selectivity to *p*-xylene reached 63% with 90% DMF conversion. Feng *et al.* designed mesoporous aerosol with sulfonic acid groups (SiO_2_–SO_3_H) as the catalyst for highly selective production of *p*-xylene from DMF and investigated the effect of active site location on catalytic performance.^96^ Toste *et al.* have developed an innovative pathway to convert DMF and acrolein to *p*-xylene for bio-renewable PET production through these steps consisting of a Diels–Alder reaction, oxidation, dehydrative aromatization, and decarboxylation. However, the process uncovered in this work would certainly not be immediately feasible because of the low temperature conditions required in the Diels–Alder reaction step and the moderate yield of the aromatization step.^90^ Recently, Tsang *et al.* reported a novel catalytic transformation of biomass-derived furans and ethylene produced *in situ* by dehydration of bio-derived ethanol to aromatics over zeolite catalysts.^97^ They demonstrated that ethanol can act as a dienophile source for furan cycloaddition instead of gaseous ethylene, leads to remarkably higher reaction rate and higher selectivity into aromatics because of lower activation barriers. Obviously, the innovative process is completely renewably and is safer to operate compared to the use of ethanol. With similar strategy, Li and co-workers reported a highly atom-economic route for the continuous production of *p*-xylene from biomass-derived building blocks.^85^ This new process uses W_2_C/AC as a robust non-noble metal catalyst to mediate a cascade dehydroaromatization and hydrodeoxygenation reactions in the absence of external redox species, providing excellent *p*-xylene yield of 90%. Notably, the new process is readily applicable to the synthesis of various (multi)methylated benzenes from bio-based building blocks.^84,85^

**Scheme 6:**
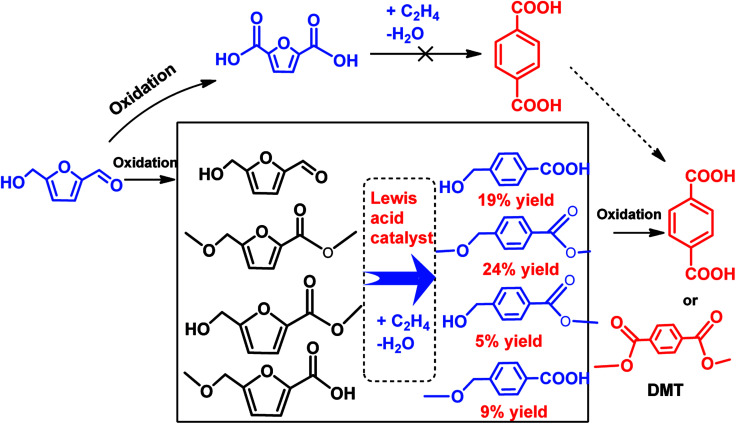
Diels–Alder pathways to PTA and DMT from HMF using oxidation steps.

In another scenario, a number of studies demonstrated successful conversion of HMF derivatives into other aromatics besides *p*-xylene.^79,83,98–102^ Davis *et al.* have developed a new alternative pathway to terephthalic acid (PTA) by the reaction of oxidized products of HMF and ethylene over solid Lewis acid catalyst.^103^ It was found that the partially oxidized HMF, 5-hydroxymethylfuroic acid (HMFCA) is reacted with high-pressure ethylene over Sn-beta to yield 4-hydroxymethylbenzoic acid with 31% selectivity at 61% HMFCA conversion after 6 h at 190 °C, as shown in Scheme 6.^103^ Moreover, no any reaction occurred between 2,5-furandicarboxylic acid (FDCA) and high-pressure ethylene even under high temperature (220 °C) and longer reaction times (14 h), showing that the strong deactivating effect of two carboxyl groups hampered the Diels–Alder reaction with ethylene.^103^

### 2.5 Catalytic conversion of HMF into amines by reductive amination

Aminofurans, especially aminoalkyfurans, are important structural units in many natural active components and are extensively used in pharmacological. Thus, the development of green and efficient strategy for the production of aminoalkyfurans starting from bio-based feedstocks has received significant attention.^104–106^ Cukalovic and Stevens developed a facile and convenient procedure for the synthesis of several novel and known aminomethyfurans starting from HMF.^107^ The production route includes a one-pot, two-step reductive amination without the purification of the intermediate imines. In this work, NaBH_4_ was used as the reducing agent, however, it is indeed not the ideal choice since NaBH_4_ can generate waste salts after reduction. Zhang and coworkers performed reductive amination of HMF with a range of anilines and secondary amines.^106^ The amino alcohols based on the anilines were obtained in excellent yields (>90%), whereas those based on the secondary amines were obtained with yields from 67% to 87%. In addition, the yield of amino alcohol reached 86% when Rh/Al_2_O_3_ was served as catalyst in the reductive amination of HMF.^108^ Esposito and coworkers reported that the reductive amination of HMF with the sodium salt of alanine using FeNi/carbon as catalyst gave excellent yield of 99%.^109^

### 2.6 Catalytic oxidation of HMF into maleic anhydride

Maleic anhydride (MA) and its derived product maleic acid (Mac) are versatile chemical intermediates with extensive applications, ranging from the production of vinyl compounds, agrochemicals, pharmaceuticals, *etc.* These two chemicals are currently produced by selective oxidation of *n*-butane.^110^ Recently, renewable approaches to produce the two compounds *via* selective oxidation of biomass-derived platforms like HMF and furfural,^110–112^ has attracted increasing attention.

V-based catalysts are found to be the most common and effective for the formation of maleic anhydride until now. It has been reported that the process for the transformation of HMF into MA afforded a MA yield of 52% and 64% over VO(acac)_2_ and vanadium-containing heteropoly acid catalysts, respectively.^111,113^ However, from the practical point of view, readily separated and recovered heterogeneous catalytic systems can decrease the production cost and therefore are more suitable than homogenous catalytic systems. Yin *et al.* explored a catalytic aerobic oxidation of HMF into maleic anhydride and maleic acid with vanadium-substituted heteropolyacid as the catalyst.^113^ Under the optimal conditions, a combined yield of 64% for maleic anhydride and maleic acid was obtained. Moreover, the mechanistic studies excluded FDCA, DFF, HMFCA, and FFCA as the reaction intermediates in the pathway of HMF oxidation to maleic anhydride. They proposed a new mechanism that the oxidation of HMF is initiated by the C–C bond cleavage between the hydroxymethyl group and furan sketch of HMF by H_5_PV_2_Mo_10_O_40_ catalyst.^113^ Recently, Li *et al.* found that a series of V-containing oxides such as VOHPO_4_, (VO)_2_P_2_O_7_ and V_2_O_5_ showed relatively good catalytic activity (more than 75% yield of MA) for the selective oxidation of HMF.^114^ They suggested that the free radical reaction could be involved in the aerobic oxidation of HMF to MA according to the case that a free radical inhibitor 4-*tert*-butylphenol could significantly reduce HMF conversion and MA yield.^114^ The recycle experiments demonstrated that V_2_O_5_ was supported on fumed silica as a heterogeneous catalyst compared to bulk V_2_O_5_, and thereby exhibiting better recyclability. In comparison, bulk V_2_O_5_ leached fast during the reaction process.^114^

**Scheme 7:**
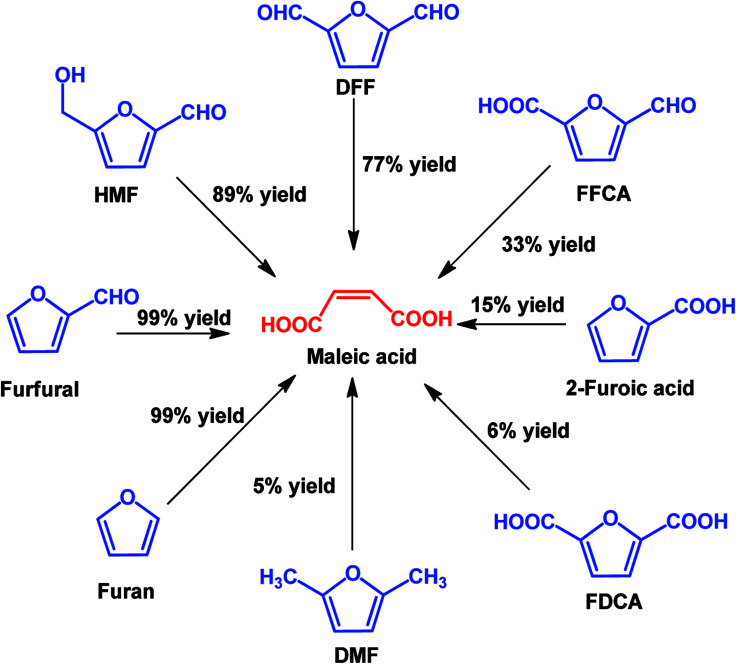
Oxidation of various furan derivatives to MA with H_2_O_2_ as the oxidant in formic acid solvent.

In view of poor catalyst recyclability of supported transition-metal oxide catalysts, Li *et al.* developed a facile metal catalyst-free system for the conversion of furfural into maleic acid with H_2_O_2_ as an oxidant and formic acid as the solvent, which obtained an unprecedented 95% MA yield.^115^ In addition, this strategy is also effective for the production of MA from other platform molecules such as HMF and HMF derivatives, as shown in Scheme 7. It can be seen that almost quantitative yield (99%) of MA was obtained from furan, and good to excellent yields of MA (77–91%) were also achieved if the furan ring was attached with aldehyde groups such as using HMF and DFF as the substrates. When the furan ring was substituted with carboxylic acid and methyl groups, lower MA yields were obtained, showing that carboxylic acid and methyl groups have a negative effect on MA production.

It is of great importance to develop continuous-flow gas phase reactions in chemical industry due to the advantages of product separation and catalyst reusability.^115^ However, the gas phase fixed-bed catalytic oxidation of furfural to MA received very little attention. V_2_O_5_/γ-Al_2_O_3_ catalyst was used in the gas oxidation of furfural into MA, but the catalyst was unavoidable deactivated by deposited of maleates and/or resin like product especially at low reaction temperature. At a given high temperature, an initial MA yield of 68% was achieved and the yield was still greater than 50% after 15 h on stream with the use of 1.0 vol% furfural and O_2_/furfural mole ratio equal to 10 at 593 K.^116^ Recently, Zhang *et al.* reported a plate vanadium phosphorus oxide as an efficient catalyst for gas phase oxidation of furfural to MA. A 90% yield of MA was achieved at furfural concentration of 10 vol% with air as an oxidant.^117^ Moreover, the catalyst exhibited good stability over the duration of 25 h of time-on-steam.

The mechanism of HMF aerobic oxidation to MA is still under debate, and some novel, robust and highly efficient catalyst need to be further explored in order to meet the large scale application. The alternative strategy with the use of renewable HMF provides a sustainable pathway to produce MA, which would alleviate the dependence on petroleum-based feedstocks.

### 2.7 Valorization of HMF into other valuable chemicals

In addition to the above noted chemicals, extensive interest has also been focused on the production of other chemicals such as caprolactam, DMTHF, DHMTHF, and levulinic acid from HMF.

The production of caprolactam (the monomer for nylon-6) or 1,6-hexanediol (monomer for high performance polyesters, polyurethane resins, and adhesives) in industry is currently a benzene-based process containing tedious seven steps. Recent elegant work by de Vries and co-workers proposed a relatively simple multistep route to caprolactam from HMF, proceeding with 2,5-bishydroxymethyl-tetrahydrofuran, 1,6-hexanediol and caprolactone as the key intermediates.^118^ It is valuable because this technology provided an overall selectivity of 86% caprolactam from HMF, meaning that the production of 1 kg of caprolactam would require 1.44 kg of HMF. This progress paves a new avenue for the transformation of renewable biomass resources to produce caprolactam, albeit the productivity of each step still needs to be further improved from the practical application viewpoint.

DMTHF is the deep reduction product of DMF, and it contains a higher energy content than DMF and may provide additional stability on storage over extended periods of time because of its fully hydrogenated furan ring.^13^ DMTF can also serve as an ideal substitute for tetrahydrofuran (THF) in chemical industry. DMTHF can be generated with HMF as an intermediate from carbohydrates, subjected to hydrolysis, dehydration and subsequently selective hydrogenation processes. Sen and co-workers provided a facile route of the one-pot conversion of carbohydrates to DMTHF.^119^ In their study, a homogenous catalytic system consisting of soluble Rh salt as a catalyst, HI and chlorobenzene as an additive, was employed in water under mild conditions. The highest DMTHF yields of 86% and 70% were achieved, with fructose and glucose as substrates in a homogenous system, respectively.^119^ Li *et al.* reported a novel process for one pot production of DMF and DMTHF from fructose by optimizing the synergic effect of Ru/C catalyst assisted by an ionic liquid. DMF and DMTHF can be produced by the dehydration of fructose followed by *in situ* hydrodeoxygenation of the resulting HMF on Ru/C catalyst with the use of ionic liquid and a biphasic [BMIM]Cl/THF solvent, and an optimal total target product yield of 67% was afforded.^120^ The above catalytic system of one-pot conversion of carbohydrates to furan based fuels features a viable process and it gives a high efficiency.

Dihydroxymethyltetrahydrofuran (DHMTHF) is commonly utilized as a precursor for the manufacture polyols and polymers or as a solvent for the conversion of carbohydrates.^121,122^ To produce DHMTHF, the aldehyde functional group and furan rings in the HMF should be completely hydrogenated without further hydrogenolysis. Thus, a desired catalyst should have suitable hydrogenation activity toward the aldehyde group and C=C double bond of furan ring and avoids the breakage of C–O and C–C to obtain high yield of DHMTHF. In 2013, Tucker *et al.* studied that HMF was completely hydrogenated over the Pd/C catalysts, achieving 90% DHMTHF yield in the saturated 1-butanol with water.^123^ Compared to monometallic catalysts, bimetallic catalysts tend to exhibit higher activity for the completely hydrogenation of HMF due to the synergistic effect between metals. For instance, when a silica-supported bimetallic Pd and Ni catalyst (Pd–Ni/SiO_2_) was applied, DHMTHF yield was up to 96.0% in water at 40 °C for 2 h under 80 bar H_2_, which was higher than that over silica-supported palladium (Pd/SiO_2_) or silica supported nickle (Ni/SiO_2_). The excellent catalytic activity of Pd–Ni/SiO_2_ was ascribed to the synergy of Pd and Ni in promoting the hydrogenation of the aldehyde group and furan ring of HMF and suppressing the subsequent hydrogenolysis of DHMTHF. To simplify the preparation pathway of DHMTHF, Yang *et al.* proposed a novel catalytic system that performed a one-pot tandem process to obtain DHMTHF directly from fructose in a biphasic system.^124^ The combination of Amberlyst-15 and silica-supported ruthenium modified by trimethylchlorosilane (Ru/SiO_2_-TM) afforded a 34% yield of DHMTHF directly from fructose in the cyclohexane/H_2_O biphasic system.

Levulinic acid is not only widely used in the production of resins and biological active materials, but also can be employed as dyes and pesticides intermediates.^125^ Traditionally, levulinic acid can be produced by the rehydration reaction of HMF catalyzed by mineral acid. To date, some novel and efficient solid acid catalysts has been applied in the production of levulinic acid, such as zeolite, solid superacid, pillared clay and others.^126,127^ In the work reported by Ramli *et al.*, Fe/HY catalyst exhibited the highest catalytic performance with 62% levulinic acid yield at 180 °C in 180 min.^126^ Dumesic and co-workers unfolded considerable results of LA production (>70%) with furfural alcohol as a starting material in monophasic THF–H_2_O medium over H-ZSM-5 catalyst.^128^ Following this research, Requies and co-workers presented the improved LA yield (77%) from furfural alcohol using ZSM-5 under an optimized reaction condition.^129^ Lourvanij and Rorrer studied a pillared clay as a solid acid catalyst for the dehydration of glucose with typical LA yields of 20%.^130^ Recently, Chen and co-workers reported the application of a solid superacid (S_2_O_8_^2−^/ZrO_2_–SiO_2_–Sm_2_O_3_), which yielded 23% of LA from rice straw at high temperature.^131^

## 3. Conclusions and outlook

An efficient utilization of renewable non-edible lignocellulosic biomass to valuable products is one of the most important challenges of mankind and is a key issue of sustainability. FDCA can be produced by chemo- and bio-catalytic oxidation of HMF, but chemo-catalysis could have some advantages over bio-catalysis due to its higher activity and readily recycling. HMF-derived amine can be generated by reductive amination of HMF over chemo-catalyst, but bio-catalyst has not yet been reported for the synthesized of HMF-derived amine. The production of aromatics using HMF as a starting material is a very promising route by Diels–alder reaction and followed by a dehydration reaction. It would be great potential providing that cost-effective commercialization of furans from biomass can be realized. DMHF can be formed by a chemo-catalytic hydrogenation over noble metal catalysts or through a bio-catalytic reduction of HMF.

Although great advances have been achieved in the transformation of HMF into valuable chemicals, it is still a big challenge to realize the commercial large-scale production of high-value chemicals from HMF in biorefineries. Future research can be focused on the following aspects: (1) although the high yield of HMF can be achieved from edible materials including fructose and glucose, it still faces tremendous problems for the transformation of non-edible lignocellulosic materials such as straw and wood to HMF, especially the use of high concentration of lignocellulose as a starting material. Thus, robust catalytic reaction system is to be developed so as to achieve a large-scale and economic production of HMF. (2) Most of the existing transformation processes generally conduct with very low substrate concentration (<10%), which lead to large-volume solvents handling and therefore increasing separation costs. Much effort should be devoted to the conversion of the substrate with high concentration. (3) The chemo-catalytic conversion of HMF into valuable chemicals may afford an efficient route to produce chemicals and bio-fuels. However, the design and development of robust heterogeneous catalysts with high activity and stability, especially under harsh conditions, need to be further explored. The life and stability of the catalyst still need to be further improved in order to meet a large-scale production. In addition, avoiding the usage of environmentally unfriendly additives in the reaction system is a trend for a green, sustainable process. (4) The bio-catalytic conversion of HMF could offer an environmentally friendly, fossil-independent alternative production pathway to produce high value chemicals. However, the low productivity, low substrate concentration, and even the diluted and large volume fermentation broth could cause extensive energy consumption for product separation and concentration in the downstream processes. (4) It is promising necessary to develop some more efficient and novel processes by integrating the bio-catalysis, chemo-catalysis even photo- and electro- catalysis in order to address some technical challenges in the valorization of biomass into value-added chemicals.

## Conflicts of interest

There are no conflicts to declare.

## Supplementary Material
